# The epidemiology, risk factors and impact of exposure on unintentional surfer and bodyboarder deaths

**DOI:** 10.1371/journal.pone.0285928

**Published:** 2023-05-18

**Authors:** Jasmin C. Lawes, William Koon, Ingvar Berg, Dion van de Schoot, Amy E. Peden

**Affiliations:** 1 Surf Life Saving Australia, Sydney, New South Wales, Australia; 2 Beach Safety Research Group, UNSW Sydney, Sydney, New South Wales, Australia; 3 School of Biological, Environmental and Earth Sciences, UNSW Sydney, Sydney, New South Wales, Australia; 4 Surfing Medicine International, The Hague, The Netherlands; 5 Emergency Department, Haaglanden Medical Centre, The Hague, The Netherlands; 6 Emergency Department, Te Whatu Ora Waikato, Hamilton, New Zealand; 7 School of Population Health, Faculty of Medicine and Health, UNSW Sydney, Sydney, New South Wales, Australia; West China Second University Hospital of Sichuan University, CHINA

## Abstract

Surfing and bodyboarding (SAB) are popular activities, but not without risk. Limited SAB mortality and exposure risk explorations exist, so this cross-sectional study explores epidemiology and risk factors for SAB deaths (1 July, 2004–30 June,2020) in Australia: including decedent and incident profiles, causes of death, differences between fatalities during SAB and other coastal activities; and the impact of exposure on SAB mortality risk. Fatality data were sourced from the National Coronial Information System, incident and media reports. Tide-state data, population data and participation data were sourced from relevant authorities. Analyses included chi-square testing and simple logistic regression with odds ratios. There were 155 SAB deaths (80.6% surfing; 96.1% male; 36.8% aged 55+years; 0.04/100,000 residents; 0.63/100,000 surfers). Drowning was the most common cause of death (58.1%; n = 90), but higher in bodyboarding, with bodyboarders 4.62 times more likely to drown than surfers (95%CI: 1.66–12.82; p = 0.003). Almost half (44.5%; n = 69; χ^2^_2_ = 9.802; p = 0.007) were with friends/family, and the largest proportion occurred during a rising tide (41.3%; n = 64; χ^2^_3_ = 180.627; p<0.001) followed by a low tide (36.8%;n = 57). Australians surf 45.7 times each year, for 1.88 hours each visit equalling 86.1 ‘exposed’ hours. With exposure-time considered, exposure-adjusted surfer mortality rate (0.06/1 million hours) is lower than other in-water activities (0.11/1 million hours). Younger surfers (14–34 years) surfed more yet had the lowest mortality rate (114.5 hours/year; 0.02/1 million hours). Older surfers (55+ years) had a lower SAB mortality rate (0.052) than the all-cause crude mortality rate of their average population counterparts (1.36). Cardiac conditions were identified in 32.9% (n = 69) of SAB deaths. SAB are relatively safe, with lower exposure mortality rates than other activities. Prevention should target older surfers, inland residents, and identification of surfers with risk factors for cardiac events.

## 1. Introduction

Surfing and bodyboarding are popular recreational coastal activities, with surfing also considered one of the oldest sports, having historical and cultural significance throughout Polynesia and communities across the Pacific ocean [1]. Today, surfing and bodyboarding (SAB) contributes significant recreational and economic value to coastal communities worldwide, including via surf tourism, the sale of surf wear and equipment and the growth of artificial wave parks [2]. Surfing and bodyboarding involve an individual riding on the face of a moving wave of water using a buoyant board (e.g., surfboard or bodyboard), which carries the rider towards the shore [3, 4]. Body surfing is also a popular activity however, for the purpose of this paper, body surfing has been excluded due to the lack of this buoyant board, although some potential injuries may be similar. Surfing and bodyboarding differ by board type and the stance of the rider; surfing generally involves standing up on a board to ride a wave while body boarding usually involves the rider laying in a prone position with the upper part of the body on the board parallel to the seafloor. In comparison to surfing, body boarders differ in wave selection with body boarding practiced in steeper waves within the impact zone of beach breaks or surf ledges with a rocky bottom [3]. Body boarders also often wear fins on their feet to increase propulsion, which improves capacity to catch waves and swimming efficiency if the board is lost.

Surfing and bodyboarding (herein referred to as ‘surfing’ unless otherwise noted) are predominantly coastal sports due to the requirement of waves, but do occur at other locations such as standing waves in rivers and increasingly in artificial surf parks where waves are generated in a controlled environment. Surfing is considered relatively safe when compared to other aquatic sports [4, 5], but still involves the potential for serious injury or death and has been identified as a high-risk activity [6, 7]. Severe surfing injuries are relatively rare [5], with the majority of surfing-related hospitalizations due to direct trauma (e.g., head injuries, cervical spine fractures, other spinal cord injuries), lacerations and drowning [8–15], with a range of morbidity outcomes. To date there has been a paucity of research into fatalities that occur while surfing and bodyboarding. This is due to studies reporting surfing-related injury capturing data via retrospective surveys or consecutive patient presentations to hospital, as well as a lack of data due to limited injury registration worldwide, with only a small number of countries represented in data collection [5, 8, 16, 17].

Drowning is a leading cause of death globally estimated to claim 295,000 lives each year [18] with many more non-fatal drowning incidents [19–22]. In high income countries such as Australia, drowning is commonly associated with recreational activities, including surfing [23]. Drowning is thought to be a leading cause of death in fatal surfing incidents [23–26] and while the increased risk of non-drowning injury has been extensively acknowledged (e.g. head or neck injury, shark attack; [4, 5, 9, 27]), there is a lack of research investigating surfing fatalities from drowning and/or other causes. Precipitating medical factors (e.g., cardiac conditions, epilepsy) are also thought to increase risk or mortality, especially when considerable levels of exertion may be required [28, 29]. Currently, the mortality rate for surfing is unknown [5], with few studies exploring surfing-related fatalities [11, 24, 26, 30].

Given surfers and bodyboarders are distinct cohorts compared to other aquatic activities, and the scant research investigating participation in surfing and the related exposure to risk of injury and death [31–34], further research is vital for informing prevention efforts. Current data estimate the number of surfers worldwide to be 23 million [5], although this has varied from 17–35 million [35–37], and 6 million bodyboarders [38]. Of the estimated 35 million surfers, America is estimated to have 13.5 million surfers, followed by Oceania with 6.5 million surfers, Asia with 6 million and Europe and Africa with 4.5 million surfers respectively [35]. Eighty-one percent of participants are estimated to be male [35], however surfing among females is gaining popularity [39, 40], as it is among people aged 50 years and older [35]. In Australia, data from the 2013/14 financial year (the most recent data available) suggests 196,000 Australians participate in surf sports (defined as surfing, bodyboarding and stand-up-paddle boarding) [41]. Other estimates indicate 2.5 million people participate in surfing, with survey data indicating surfers self-report spending an average of anywhere from 3–11 hours per week in the water [23, 30, 42].

Australia records, on average, 283 fatal unintentional drownings each year [43], of which an average of 112 occur in the coastal and ocean environment [23]. Surf Life Saving Australia report on coastal and ocean drowning annually, including watercraft-related fatalities which includes incidents occurring while surfing and bodyboarding. However, to date there has been no specific analysis of surfing and bodyboarding-related mortality, or exploration of risk factors, that considers the issue of exposure.

Motivated by a lack of research in this space, both in Australia and globally, this study characterizes the epidemiology and risk factors of unintentional death while surfing and bodyboarding in Australia. In addition, this study uses participation data as an alternative population to assess the impact of exposure on risk. Our primary aims were to (1) describe the decedent and incident profile of surfing and bodyboarding fatalities, including situations with multiple causes of death; (2) explore mortality burden and risk through total population, exposed population and person-time calculated death rates; (3) evaluate variation in types of surfing and bodyboard-related fatalities comparing drowning and other non-drowning causes of death; and (4) assess differences between surfing fatality profiles and those of other in-water recreational coastal activities.

Specifically, we hypothesize that (1) surfer and bodyboarder mortality are relative to participation levels, (2) surfer and bodyboarder mortality risk changes when exposure time is considered; (3) drowning is the dominant cause of death; and (4) surfing and bodyboarding is generally safer than other coastal activities.

## 2. Material and methods

This study is a population-based cross-sectional analysis of unintentional fatalities among surfers and bodyboarders who died while engaged in surfing activities in the Australian coastal environment between July 1, 2004 and June 30, 2020. This period was chosen due to data availability, fiscal year reporting periods (July–June), and ethical agreements governing the period of mortality data collection.

### 2.1 Data sources and variables

The National Coronial Information System is an electronic database of all deaths notified to coroners within Australian and New Zealand. The information held within this database is sourced from the Victorian Department of Justice and Community Safety and includes coroner’s findings, police narratives, autopsy and toxicology reports, and is considered the data with the highest level of validation and rigour for epidemiological studies within Australia. Information on surfing and bodyboarding fatalities were collated into Surf Life Saving Australia’s fatality database from the National Coronial Information System, media reports and Surf Life Saving Australia’s SurfGuard Incident Report Database as per previous studies [44–46]. All unintentional surfing and bodyboarding deaths that occurred in Australian coastal waters (i.e. including up to 3 nautical miles offshore) between 1^st^ July 2004 and 30^th^ June 2020 were collated for analysis. Both open (i.e., under coronial investigation) and closed coronial cases were included in the analyses, although at the time of analyses 92% were closed (n = 144). Only unintentional deaths of people engaged in surfing or bodyboarding were included; fatalities which occurred while body surfing or when using other unpowered watercraft such as canoes, kayaks, jet skis, windsurf boards, and kite surfing boards and stand-up paddle boards were excluded as their activity mechanics, safety practices and prevention strategies are different (e.g. lifejacket usage is recommended).

Coroner determined cause of death can span one primary cause or may also include up to five additional contributory causes of death (i.e., cause 1a [primary cause of death], 1b, 1c, 1d, 2 and 3). As the Surf Life Saving Australia fatality database is primarily geared towards informing prevention efforts and since drowning as a cause of death is generally determined using a method of exclusion [47], all cases where drowning or immersion was the primary (cause 1a) or a contributory cause of death (cause 1b-3) were classified as drowning. Cause of death remained undetermined or unascertained in eight cases (5.2%): for six of these cases, the coroner could not exclude drowning or immersion, other data sources indicated a drowning event, and a consensus process with drowning researchers determined a submersion fatality. The remaining two undetermined cases were classified as ’other’ fatalities due to details within each incident’s narrative. Contributing causes of death (i.e., causes 1b-3) were grouped and broadly classified as cardiac, neck/head injury, shark or lightning, and other medical or trauma.

Data on the presence of rip currents and craft type (surfboard or bodyboard) were sourced from the narratives reported within coronial, police, autopsy, incident, and media reports. Data on tide state were sourced from Surf Life Saving Australia’s Fatality Database in which weather and ocean data from the Bureau of Meteorology are linked to each incident and were coded as either occurring during hours of rising tide, high tide, dropping tide, or low tide. Time of day was coded using incident time into: Morning (6:01am to 12pm); Afternoon (12:01pm to 6pm); Evening (6:01pm to 6am) or Unknown (for cases where incident time was unknown). Seasons in Australia are coded as follows: Summer (December to February); Autumn (March to May); Winter (June to August); and Spring (September to November). Weekdays included incidents that occurred between Monday to Friday, and weekend incidents occurred on Saturday and Sunday.

Using data from Surf Life Saving Australia’s fatality database, we compared the fatality risk while surfing and body boarding to other in-water recreational coastal activities (i.e., swimming/wading, snorkelling, diving and using other watercraft [i.e., canoes, kayaks, stand up paddleboards]). These common activities were chosen as they are not externally powered (as with boating or personal watercraft/jet skis), often require the participant to intentionally enter the water (except for some watercraft) and can be exposed to similar environmental hazards as surfing and bodyboarding activities.

Population and all cause mortality data were sourced from the Australian Bureau of Statistics (2004–2020) [48], and surfing exposure and participation estimates were derived from Surf Life Saving Australia’s National Coastal Safety Survey (2014–2020). The National Coastal Safety Survey is described in detail elsewhere [31], but briefly is a nationally representative survey conducted with Australian residents aged 16 years and above (overall N = 10567 respondents, annual mean $\bar{x}$ [95% CI] = 1510 [±89.9] respondents). The initial survey target population was 16–69 years (2014–2017, approximately n = 1,400), but this was extended to 16 years of age and above from 2018 (approximately n = 1,600 for 2018–2020) [31]. This was important to produce results that were more representative of the population, and to develop a more accurate understanding of coastal visitation and participation. This change was made for practical reasons, as prior to 2018, it was understood that internet penetration was relatively poor in older Australians (70+ years of age) and would not reflect the actual population, but now is considered appropriate for accurate data collection for Australians aged 35 years and older [49]. To account for this longitudinal underrepresentation in the 70+ age group, their responses were post-weighted, and increased by 1.46 times. For this study, we included respondents who reported having surfed at least once in the previous 12 months (overall N = 947 [2014–2020]). This equated to an annual average of 146 surfers and bodyboarders combined ($\bar{x}$ [95% CI] = 146 [±22.5]).

### 2.2 Statistical analysis

Descriptive statistics were used to describe the person and incident profiles of the cohort of surfing fatalities. To explore total population burden, resident-based death rates were calculated (excluding short-term international visitors) by activity (surfing vs other in-water coastal recreational activity), incident type (drowning vs other cause of death), age group (14–34 years, 35–54 years and 55+ years), sex (male and female), and residence distance to the coast (up to and including 50km and above 50km). Crude annual (financial years) and cumulative mortality rates per 100,000 Australian residents were calculated from Australian Bureau of Statistics population data. The proportion of the population who live within 50km of the coast is conservatively estimated at 85% in line with previous publications [50, 51], while the number of survey respondents who lived within 50km and further than 50km was determined within the survey. To explore exposed population burden, cumulative mortality rates per 100,000 exposed population were calculated using the estimated percent of the Australian population that surfs at least once per year, derived from National Coastal Safety Survey 2014–2020 data and applied to the entire study period [31], as the denominator. Since the National Coastal Safety Survey is conducted on Australian residents aged 16 years and above, short-term international decedents were excluded from exposure analyses. Decedents aged <16 years of age, however, were included, as this impact was expected to be minimal since >99% of cases were aged 16+. Participation data from the National Coastal Safety Survey are not available for surfing and bodyboarding separately [31], only combined fatality data were used for these analyses. Mortality rates per 1 million surfing hours were also calculated using National Coastal Safety Survey participation data: the estimated number of annual participation hours (denominator) was derived by multiplying the frequency of activity-based coastal visits (using the number of responses within seven weighted categories ranging from *less than 3 times* a year to *every day*) by the average length of time spent participating in the activity (using the number of responses within seven categories ranging from *15 minutes* to *5 hours or more*). Population and cause of death data were downloaded from the Australian Bureau of Statistics [52, 53] by age group and sex for the study period and used to calculate crude mortality rate and exposure adjusted mortality rates.

Simple logistic regression with odds ratios was used to test for differences between drowning and other causes of surfing related death; separate models were constructed with single fixed effect terms to evaluate if age, sex, craft type, presence of a rip current, season, day of the week, if the victim was alone, distance between residence and drowning location, visitor status, or toxicology status were statistically associated with type of death. With Bonferroni correction, statistical significance for logistic regression was set at 0.005 [54]. Non-parametric chi-square testing was used to assess for differences in the age and sex proportions of surfer and bodyboarder deaths compared to the Australian population, proportion of deaths by craft type (surfboard vs bodyboard) compared to estimated global surfboard and bodyboard users [35, 38], and the proportion of deaths by season, weekday, and time of day. Additionally, non-parametric chi-square tests were used to evaluate for differences in the proportion of deaths among surfers and bodyboarders compared to other in-water coastal recreational activities (defined as swimming/wading, snorkelling, diving, and other watercraft) across a range of variables. A modified Bonferroni correction was applied where multiple variables within a single test were analysed [54], deeming statistical significance at 0.01 for non-parametric chi-square tests.

Descriptive and chi-square analyses were performed in SPSS V26 [55], logistic regression was conducted in R [56]. This study was conducted with ethics approval from the Department of Justice and Community Safety Human Research Ethics Committee (JHREC; CF/07/13632; CF/10/25053; CF/14/1169; CF/16/17314), and survey data has been approved for use by University of New South Wales (UNSW) Sydney Human Research Ethics Committee Panel B: Arts, Humanities & Law (HC200950). No participant consent was obtained for fatality data as they were deceased. The anonymised survey data was obtained via an independent third party to which participants subscribe.

## 3. Results

**Fig 1:**
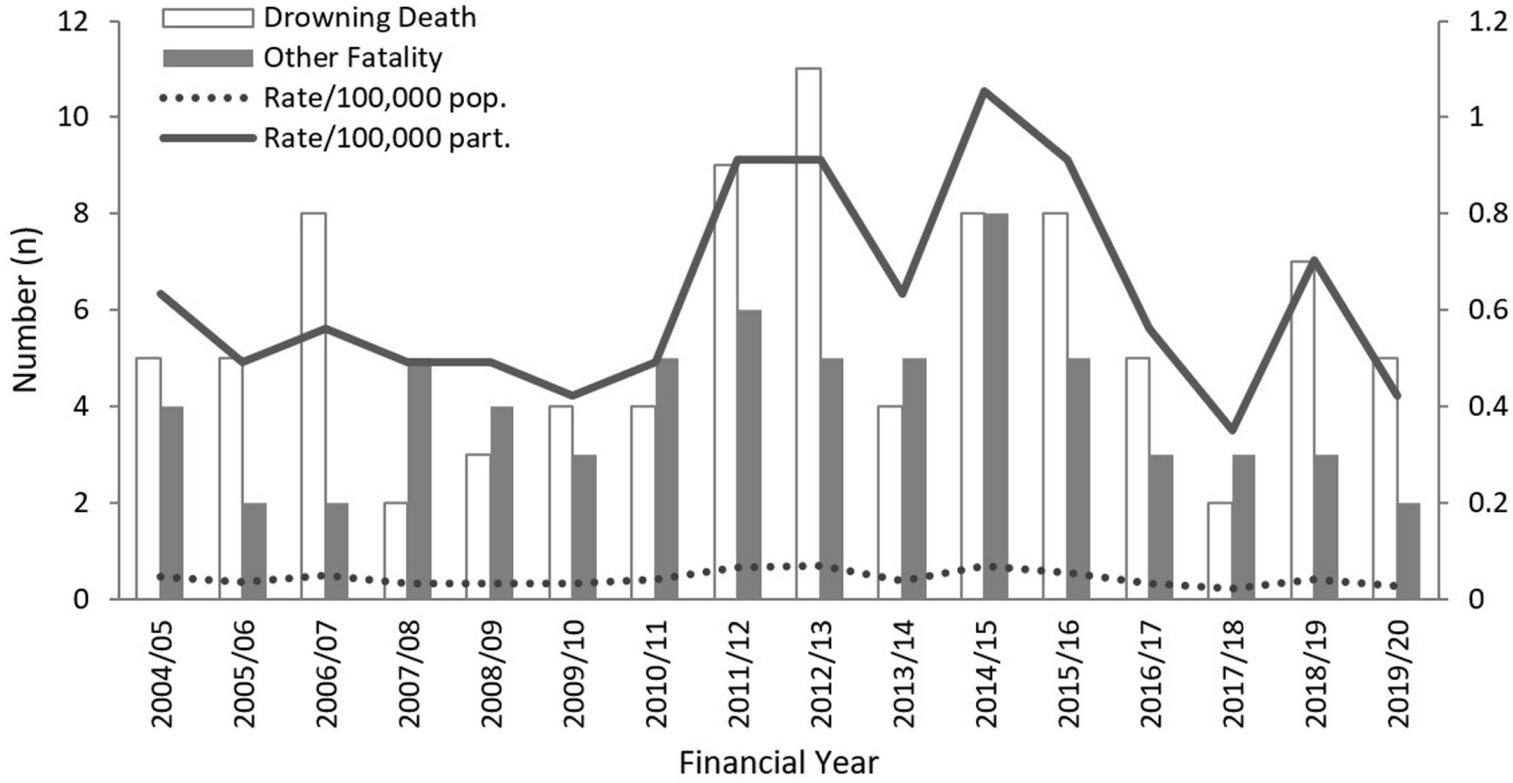
Surfing and bodyboarding deaths over time by incident type. Mortality rates have been calculated using the Australian population (black) and using average surfing and bodyboard participants aged 16 years and older (dotted line).

Between July 1, 2004 and June 30, 2020, 155 people died while surfing or bodyboarding in Australia. The person and incident profiles of surfer fatalities are distinct from what would be expected from the general population (Table 1) and compared to other coastal in-water recreation activities (Table 2). While the number and rates of surfing and bodyboarding deaths has fluctuated over time (Fig 1), an average of 9.7 (SD = 3.37) surfing and bodyboarding-related fatalities occurred per year (9.1 excluding short-term visitors), the cumulative resident-based mortality rate (resident n = 143) was 0.04 per 100,000 residents. The mean annual duration surfing exposure per surveyed surfing and bodyboarding participant (n = 974) was 86.1 hours, estimated from an average of 1.88 hours spent surfing from 45.7 surfing visits. Generalising these results to the exposed surfing and bodyboarding resident population, this exposure-adjusted mortality rate equates to 0.63 per 100,000 per estimated surfing and bodyboarding participating residents. Death rates per 100,000 Australian residents, 100,000 participating Australian residents and per 1 million activity hours are presented in Table 4. See also Table 5 for a comparison between SAB mortality rates and all-cause mortality rates by age and sex.

**Table 1:** Non-parametric analyses of observed proportions of the person and incident profiles of surfer and bodyboarder fatalities compared to expected proportions from various sources. Further statistical details of expected models are denoted for each variable and described as table footnotes. **Bold type** indicates significance, deemed at p = 0.01 after Bonferroni correction.

Variable	Category	N	%	Expected N	Expected %	p value
Age group	14–34 years	37	23.9			
	35–54 years	49	31.6			
	55+ years	57	36.8			See Table 4
Sex	Male	140	90.3			See Table 4
	Female	3	1.9			
Craft type**	Surfboard	125	80.6	23 million	79.3	X^2^_1_ = 0.168
	Bodyboard	30	19.4	6 million	20.7	p = 0.682
Tide state^#^	Rising Tide	64	41.3	10 hours	41.7	X^2^_3_ = 186.806
	High Tide	17	11.0	2 hours	8.3	**p<0.001**
	Dropping Tide	17	11.0	10 hours	41.7	
	Low Tide	57	36.8	2 hours	8.3	
Season ^^^	Summer	40	25.8	90.25 days	24.7	X^2^_3_ = 2.147
	Autumn	45	29.0	92 days	25.2	p = 0.543
	Winter	38	24.5	92 days	25.2	
	Spring	32	20.6	91 days	24.9	
Weekend ^^^	Weekday	101	65.2	5 days	71.4	X^2^_1_ = 2.983
	Weekend	54	34.8	2 days	28.6	p = 0.084
Time of day^#^	Morning (6am-12pm)	79	51.0	6 hours	25.0	X^2^_2_ = 128.844
	Afternoon (12pm-6pm)	68	43.9	6 hours	25.0	**p<0.001**
	Evening (6pm-6am)	7	4.5	12 hours	50.0	
	Unknown	1	0.6			
Residence distance from anywhere on the coast*	Within 50km (0-50km)	138	89.0	15278608^a^	85.0	X^2^_1_ = 16.531
	Above 50km	4	2.6	2696225^b^	15.0	**p<0.001**
	International*	10	6.5			
	Unknown*	3	1.9			

* Expected proportions calculated using Australian population aged 14–85 from the Australian Bureau of Statistics [48], observed numbers included those who resided in Australia and excluded international and short-term visitors)

** Expected proportions calculated using global estimates of surfing and bodyboarder participant populations [35, 38]

^a^ Expected proportion estimated as 85% of residents of surfing population living within 50km from the coast [50, 51]

^b^ Expected proportion estimated as 15% of resident of surfing population living greater than 50km from the coast (100%-85% = 15% mentioned above in ^a^)

^#^ Calculated using the average duration (hours) within a 24-hour period for each categorical variable.

^^^ Calculated using the average number of days for each period (i.e., week or season)

**Table 2:** Comparative chi-squared analyses of surfing and bodyboarding deaths to other in-water coastal activity deaths (swimming/wading, snorkelling, diving, and other watercraft, excluding surfing and bodyboarding). **Bold type** indicates significance, deemed at p = 0.01 after Bonferroni correction. Unknown cases were excluded from analyses and are denoted with an asterisk (*).

		Surfers and bodyboarder deaths		Other coastal activity deaths		Test statistic
Variable	Category	N	%	N	%	p value
	Total	155	100	939	100	
Incident type	**Drowning**	**90**	58.1	723	77	X^2^_1_ = 31.367
	**Other fatality**	**65**	41.9	216	23	**p<0.001**
Age group	14–34	37	23.9	317	33.8	X^2^_2_ = 1.633
	35–54	49	31.6	291	31	p = 0.172
	55+	57	36.8	331	35.3	
Sex	Male	149	96.1	803	85.5	X^2^_1_ = 14.094
	Female	6	3.9	136	14.5	**p<0.001**
Alone	Yes	29	18.7	131	18.9	X^2^_2_ = 9.802
	**No, with strangers**	**41**	26.5	138	61.2	**p = 0.007**
	**No, with friends/family**	**69**	44.5	425	19.9	
	Unknown*	16	10.3	245		
Visitor Category	**Resident**	**141**	91	666	78	X^2^_2_ = 17.990
	**Short Term Visitor**	**10**	6.5	145	17	**p<0.001**
	Student/Working holiday	2	1.3	43	5	
	Unknown*	2	1.3	85		
Resident status	**Resident**	**141**	91	666	78	X^2^_1_ = 17.897
	Non-resident	12	7.7	188	22	**p<0.001**
	Unknown*	2	1.3	85		
Distance between home and incident location	**Local (<10km)**	**65**	41.9	240	27.1	X^2^_3_ = 26.449
	Resident (10-50km)	29	18.7	180	20.3	**p<0.001**
	**Domestic (>50km)**	**42**	27.1	312	35.2	
	**International**	**11**	7.1	154	17.4	
	Unknown*	8	5.2	53		
Tide state	Rising Tide	64	41.3	343	43.3	X^2^_3_ = 180.627
	High Tide	17	11	68	8.6	**p<0.001**
	**Dropping Tide**	**17**	11	315	39.7	
	**Low Tide**	**57**	36.8	67	8.4	
	Unknown*	0	0	146		
Season	**Summer**	**40**	25.8	445	47.4	X^2^_3_ = 60.359
	Autumn	45	29	220	23.5	**p<0.001**
	**Winter**	**38**	24.5	83	8.8	
	Spring	32	20.6	190	20.3	
	Unknown*	0	0	1		
Weekend	Weekday	101	65.2	533	58.6	X^2^_1_ = 2.774
	Weekend	54	34.8	377	41.4	p = 0.096
	Unknown*	0	0	29		
Time of day	**Morning (6am-12pm)**	**79**	51	258	29.9	X^2^_2_ = 36.402
	**Afternoon (12pm-6pm)**	**68**	43.9	495	57.4	**p<0.001**
	Evening (6pm-6am)	7	4.5	110	12.7	
	Unknown*	1	0.6	76		
Rip current present	Yes	**19**	12.3	250	35.9	X^2^_1_ = 17.451
	No	**93**	60	446	64.1	**p<0.001**
	Unknown*	43	27.7	243		
Toxicology	Alcohol and/or Drugs	23	14.8	139	17.7	X^2^_1_ = 0.182
	None	118	76.1	647	82.3	p = 0.669
	Unknown*	14	9	153		

Drowning was listed as a cause of death in most fatalities (n = 90, 58.1%), while 41.9% (n = 65) were due to other causes (Table 2), many listed were cardiac-related problems (Table 3). While the vast majority were male (96.1%; n = 149), no evidence of a relationship between sex and cause of death (drowning vs. non-drowning) was identified (Female OR = 3.76, 95%CI: 0.41–33.01, p = 0.231).

**Table 3:** Surfing and bodyboarding causes of death by age group drawn from coronal ICD-10 coding. Sum of total numbers are greater than total number of deaths as some cases listed multiple causes of death, this is denoted with an asterisk(*).

			Age groups		
Causes of death groups	Total		14–34 years	35–54 years	55+ years
	N*	% of all fatalities (n = 155)	N (% of cause of death group)		
Drowning	90	58.1	31 (34.4%)	24 (26.7%)	35 (38.9%)
Cardiac	65	32.9	3 (4.6%)	30 (46.2%)	32 (49.2%)
Head/neck/spinal injuries	24	12.9	6 (25%)	7 (29.2%)	11 (45.8%)
Shark or lightning	13	9.0	9 (69.2%)	3 (23.1%)	1 (7.7%)
Other medical (e.g., epilepsy, stroke)	12	1.3	4 (33.3%)	4 (33.3%)	4 (33.3%)

* Sum is greater than 100% as some cases listed multiple causes of death

About one third (n = 48, 31%) of cases listed multiple causes of death, the most common combination of these (n = 25) was drowning and cardiac causes listed together (Table 3). Drowning or submersion was listed as the first cause of death in 72 cases in the National Coronial Information System and a contributing cause of death in eight cases. For ten cases, drowning was determined the most likely cause of death from other data sources when National Coronial Information System cause of death information was missing. A cardiac cause of death was listed first in 45 of the 65 cardiac cases (69.2%), and most cases with cardiac causes of death were men over the age of 45 (n = 54, 84.3%; Table 3). More than half (n = 14, 58%) of the 24 cases with a head or neck injury also listed drowning as a cause of death, and ten of the 12 cases (83.3%) with other medical or trauma also listed drowning as a cause of death. The proportion of fatalities with head/neck injuries were the same for those using a surfboard (15.2%) vs those on a bodyboard (16.7%).

Surfing-related deaths among Australian residents differed by age group relative to the population (χ^2^_2_ = 11.427; p = 0.003; Table 1) with more deaths in older age groups (55+ years; 36.8%, n = 57) and fewer in younger (14–34 years; 23.9%, n = 37). Deaths by craft type also differed by age group (χ^2^_2_ = 13.391; p = 0.001). There were no differences between age and cause of death (drowning vs. other; Logistic regression OR: 0.99; 95%CI: 0.97–1.01; p = 0.284).

Surfboards were involved in more deaths than bodyboards (80.6% vs. 19.4%), but this was proportionate to reported surfing and bodyboarding populations (Table 1). However, the proportion of deaths due to drowning compared to other causes was higher for bodyboard-related deaths compared to incidents involving surfboards, with bodyboarders 4.62 times more likely to die from drowning vs. non-drowning causes than those on a surfboard (95%CI: 1.66–12.82; p = 0.003). This relationship remains after controlling for age (age adjusted OR = 4.51, 95%CI: 1.61–12.57, p = 0.03). Surfing and bodyboarding deaths occurring during a particular tide state differed in expected proportion of hours available (Tables 1 and 2), with proportionally fewer occurring for the duration (i.e. the number of hours) that the tide was dropping (11.0%; n = 17), and significantly more than expected for the duration of the two hours of low tide (low tide: 36.8%; n = 57; Table 1) and compared to other aquatic coastal activities (Table 2). Conversely, surfing and bodyboarding deaths were less likely to involve a rip current when compared with other aquatic activities (Table 2).

Surfing deaths occurred throughout the year (Table 1), but were more prevalent in winter when compared to other coastal activities (Table 2). A similar number of deaths occurred in the morning (51%, n = 79) and afternoon (43.9%, n = 68), and less overnight (χ^2^_3_ = 128.844; p<0.001; Table 1). There was no evidence that season, weekday, or time of day were associated with a drowning vs. non-drowning death (season p = 0.8; weekday p = 0.252; time of day p = 0.454).

A greater of proportion of surfing-related deaths were recorded in the company of strangers when compared to other coastal activities, and a smaller proportion when in the company of friends or family (Table 2). There was no evidence that surfers who died alone were more or less likely to drown or die from other causes (OR = 1.09, 95%CI: 0.47–2.53, p = 0.839). Alcohol and drugs were reported as a contributing factor in 14.9% (n = 23) of incidents (Table 2); but there was no relationship between type of cause of death (drowning vs. other) and alcohol/ drug use (OR = 1.656., 95%CI = 0.634–4.325 p = 0.302).

A greater proportion of surfing-related deaths than expected from population statistics involved decedents who lived within 50km of the coast (Table 1), the highest number involved locals who lived within 10km from the incident location (41.9%, n = 65; Table 2). Surfers who lived within 50km recorded the greatest proportion of incidents and higher population-based mortality rates (Tables 1 and 4) however, the time exposure-adjusted mortality rates were higher for individuals who lived greater than 50km (Table 4). Individuals residing further away from the incident location were more likely to drown than die from other causes (trend test OR for distance categories = 1.72, 95%CI 1.21–2.44, p = 0.003).

**Table 4:** Data and calculations used to explore estimated mortality rates per 100,000 resident and exposed populations and per one million hours of participation per participant.

	Variable	Annual deaths*	Resident population**	Crude mortality rate	Population (%, 16+) who participated in previous 12 months	Exposed population	Exposure-adjusted pop. rate	No. visits/ participant/yr***	Hours/ visit***	Hours exposed/year/ participant***	Exposed-adjusted hours	Exposure-adjusted rate/ 1 million hours
**Activity**	SAB	9.1	17974833	0.051	9.8	1761534	0.52	45.70	1.88	86.1	151605630	0.06
	Other in-water activities	58.7	17974833	0.327	76.4	13732772	0.43	23.25	1.72	40.0	549815718	0.11
**Incident type**	Drowning death	5.0	17974833	0.050	9.8	1761534	0.28	45.70	1.88	86.1	151605630	0.03
	Other fatality	4.1	17974833	0.040	9.8	1761534	0.23	45.70	1.88	86.1	151605630	0.03
**Age group**	14–34 years	2.3	6727102	0.034	15.6	1049428	0.22	55.99	2.04	114.5	120136636	0.02
	35–54 years	3.1	6602859	0.047	8.8	581052	0.53	45.33	2.00	90.5	52577263	0.06
	55+ years	3.6	6988955	0.052	3.8	265580	1.36	54.41	1.46	79.4	21089990	0.17
**Sex**	Male	8.9	8996907	0.099	13.1	1175658	0.76	55.38	1.96	108.3	127341056	0.07
	Female	0.2	9080604	0.002	6.5	591989	0.03	42.52	1.80	76.5	45274045	.004
**Residence to coast (km)**	Within 50km (0-50km)	8.6	15278608^a^	0.06	8.0^a^	1226292	0.70	51.55	1.88	96.7	118555663	0.05
	Above 50km	0.25	2696225^b^	0.01	1.8^b^	47826	0.52	20.97	1.95	41.0	1959935	0.13

* Annual average including decedents aged between 14–80 years only and those who were considered residents of Australia only

** Annual average population of Australian residents aged between 14–80 years only (July 2004-June 2020)

***Average annual percentage of Australian adult population (16 who participate in surfing and other in-water coastal activities and the hours spent participating derived from data collected in Surf Life Saving Australia’s National Coastal Safety Survey 2014–2020 [57]. NB: With no better data available, this data has been applied across the entire study period, under the assumption that there were no significant deviations from this percentage of exposed population.

^a^ Estimated as 85% of resident or surfing population [50, 51]

^b^ Estimated as 15% of resident or surfing population (100%-85% = 15% mentioned above in ^a^)

According to the National Coastal Safety Survey, approximately 9.8% of the Australian population (estimated 1,761,534 residents) surf each year. Estimated mortality rates calculated for this exposed population (surfing participants) ranged from 1.3–56.4 times ($\bar{x}$ = 14.39; SD = 15.37) higher than those calculated using the total population (Table 4). On average, Australian surfers spend 86.1 hours surfing each year (Table 4), which is considerably higher than the 40.0 hours of annual participation for other in-water coastal activities. However, the exposure-adjusted mortality rate for surfing is 0.06 per 1 million hours, half that of the other in-water coastal activities (0.11 per 1 million hours; Table 4). Similarly, younger surfers (14–34 years old) spent the most time surfing (114.5 hours each year) and had the lowest exposure-adjusted mortality rate (0.02 per 1 million hours) compared to older surfers (55+ years) who were exposed less (79.4 hours each year) but had the highest exposure-adjusted mortality rate (0.17 per 1 million hours; Table 4). Surfers who lived within 50km of the coast surf 51.55 times each year, for 1.88 hours each visit, equating to 96.7 hours exposed annually (Table 4) and a relatively low exposure-adjusted mortality risk of 0.07 per 1 million hours. This is considerably greater exposure time than those who live more than 50km from the coast, who spent on average 41.0 hours each year surfing but had a higher exposure-adjusted mortality rate of 0.13 per 1 million hours (Table 4).

The crude SAB mortality rate for older people (55+ years) was 1.5 times greater than the 14–34 year-old SAB mortality rate and 1.1 times greater than 35–54 year old SAB mortality rate. When compared to population-based all-cause mortality rates, older people recorded a rate that was 30 times higher than 14-34-year-olds and 8.6 times higher than 35–54 year-olds. The mortality rate for males was 49 times greater for SAB deaths compared to 1.5 times greater for all-causes, when compared to females (Table 5).

**Table 5:** Detailed comparison of age- and sex- specific mortality rates and 95% confidence intervals of SAB deaths and Australian deaths from all causes.

	Variable	Average SAB deaths (N/year)	Crude SAB mortality rate		Exposure-adjusted rate		Average all-cause deaths (N/year)	Crude all-cause mortality rate	
			Rate	95% CI	Rate	95% CI		Rate	95% CI
**Age group**	14–34 years	2.3	0.034	(-0.010–0.078)	0.22	(-0.064–0.502)	3036	47.77	(46.072–49.470)
	35–54 years	3.1	0.047	(-0.005–0.099)	0.53	(-0.060–1.127)	10336	165.68	(162.486–168.874)
	55+ years	3.6	0.052	(-0.002–0.106)	1.36	(-0.045–2.756)	77808	1432.60	(1422.529–1442.662)
**Sex**	Male	8.9	0.099	(0.034–0.164)	0.76	(0.260–1.254)	53934	601.24	(596.162–606.311)
	Female	0.2	0.002	(-0.007–0.011)	0.03	(-0.114–0.182)	37357	410.60	(406.438–414.765)

## 4. Discussion

Surfing and bodyboarding are popular recreational coastal activities, participation in which are not without risk of injury and death [1]. This total population study from Australia, is one of the first studies to explore mortality among surfers and bodyboarders and is also the first to consider and demonstrate the impact of exposure on mortality risk. Key findings with a focus on informing strategies to reduce mortality risk while surfing and bodyboarding are now discussed.

Surfing and bodyboarding are relatively safe activities compared to other in-water coastal activities as seen in both this study and in comparison to fatal and non-fatal drowning rates based on exposure via swimming, fishing or rock fishing [33, 58]. The crude resident-based mortality rate for other in-water coastal activities was six times greater than that for surfing, although those who surf spend more than twice as many hours participating in the activity each year. While it may not be surprising that surfers die at much lower rates compared to other coastal activities, efforts to understand why is worthy of future investigation. Surfers are likely to be more familiar with surf zone dynamics and to possess a distinctly more advanced ocean skill set than other coastal visitors. Additionally, the fact that surfing as an activity incorporates a significant flotation device may further serve to reduce mortality risk, especially since the provision of flotation is a critical step in the drowning chain of survival [59]. Different from some other water-based activities, surfers and bodyboarders are generally attached to their board by a leg rope or leash, which can also act as a buoyancy aid if required. Furthermore, many bodyboarders use fins to propel themselves in the surf which may also assist in self-recovery when in trouble in the water.

Local surfers and bodyboarders had higher mortality rates compared to non-local surfers and compared to participants in other aquatic coastal activities and a greater proportion of surfing and bodyboarding decedents lived closer to the coast than would be expected from the population distribution. Local surfers spend more time surfing than those surfers who lived further away, as do surfers compared to participants of other aquatic activities and are potentially more likely to take part in their activity locally, and therefore have a greater familiarity with, and understanding of, local coastal conditions. This is more evident when we explore the time exposure-adjusted mortality rates. For example, surfers who lived closer to the coast spent more time surfing (96.7 hours each year on average) yet had a much lower mortality rate (0.06/1 million hours) compared to surfers who lived greater than 50km from the coast who, despite significantly lower exposure, reported higher exposure-adjusted mortality rate (0.13/1 million hours). The exposure-adjusted mortality rate for surfers that lived more than 50km from the coast was 56 times greater than for those who lived closer to the coast. This could be due to poor strength and conditioning [60], may highlight the risks posed by unfamiliar locations [61], or could reflect a lack of coastal safety knowledge and awareness [34]. These results demonstrate that coastal safety knowledge is an important component for water safety education, regardless of an individuals’ proximity to the coast.

This study presents multiple exposure measurements which has enabled novel perspectives on mortality risk spanning surfer and bodyboarder mortality and populations at risk. For example, younger surfers (aged 14–34 years) spent the most time surfing (114.5 hours each year on average) yet had the lowest mortality rate (0.02/1 million hours), especially when compared to older surfers (aged 55 years and over) who were exposed less but still had the highest annual number and exposure-adjusted mortality rate (0.17/1 million hours). However, when the population mortality rates were compared to all-cause deaths, the risk of death for surfers and bodyboarders is not equivalent to broader mortality patterns in general. For example, the mortality rates of older surfers were higher than other age groups, but not to the same extent as would be expected for all-cause mortality. This result suggests that surfing as an activity may be in some ways protective for older surfers, as well as supporting previous research that identifies young men as overrepresented in coastal fatality data [44]. Comparison with all-cause mortality data also suggests SAB mortality risk is spread across the surfing population, including among surfers aged 35–54 years. This highlights that surfing safety initiatives must be extended beyond typical high-risk populations or groups, with relevance to the whole surfing population. Further investigations are required to understand and identify high-risk populations or age groups to develop specific or targeted prevention efforts. Given the high incidence of cardiac events, further research is warranted to explore the cardiovascular profile and the need for screening of risk factors for ischaemic and non-ischaemic cardiac disease among surfers.

The higher mortality risk among older surfers may be related to age-related risk factors and precipitating medical factors, although it should be noted that older compared to younger surfers had a lower SAB mortality rate than for all-cause mortality rate among the same aged and general population. However, almost half of all deaths with a cardiac-related cause of death occurred in those 55 years of age and older (49.2%), and injuries to the head, neck and spine were also prevalent in this age group (45.8%). Injuries and trauma related causes of death may be due to larger bodyweights, resulting in greater impact when hitting sand banks, a higher risk of intracranial bleeding when using anticoagulants and antiplatelet drugs, or may also be due to bones being less malleable and more likely to break due to age-related factors [62]. As a high-risk group in which participation in surfing is increasing [35], it is vital to encourage all surfers and bodyboarders to undergo regular medical checks and to discuss the risks of surfing, specifically in relation to their cardiac health, with their doctor. Older surfers have been shown to reach a significantly higher maximum heart rates as a percentage of their age predicted maximum heart rate, compared to younger surfers [63]. This could cause myocardial ischemia in the presence of significant coronary artery stenosis or rhythm disorders related to exercise. Even so, all surfers regardless of age should be encouraged to always surf with, or in the vicinity of, other people, never alone. Additional strength and conditioning could be conducted, in conjunction with surfing, to negate age-related changes such as decreases in muscle mass, balance, coordination, reflexes, and agility [60].

Surfers represent an important form of bystander rescue or supervision to others in the water around them [64–66]. This has been attributed to their generally high levels of fitness and swimming competency, the fact that they have a flotation device with them, and their knowledge of surf dynamics [64, 66]. More importantly, at unpatrolled locations or at times outside of patrol periods, surfers may represent one of the only forms of assistance at the time of an incident [64, 66]. Surfers with more experience, or those who had previously rescued someone, were more likely to conduct a rescue [64], with research suggesting that Australian surfers conduct a similar number of rescues to surf lifesavers and lifeguards. The majority of surfers felt their rescue had saved a life [65, 66]. Given the role surfers play as bystander rescuers, basic lifesaving and CPR training should be strongly promoted within the surfing community [65, 66] as a measure to reduce drowning of other surfers and the general public.

Our results showed that hours of low tides presented the greatest mortality risk to surfers and bodyboarders in proportion to their duration, which is consistent with recent coastal drowning research [67]. Low tide, via changes in coastal geomorphology, can expose other coastal hazards (e.g. rocky reefs, sand bars, etc.) that can contribute to injury and risk–especially for those who may be less familiar with the locations (i.e. visitors) [68]. While this study explored impacts of rip currents and tide states, further research is required to gather and incorporate more comprehensive data on other environmental conditions. This includes data on swell size, swell period and direction, wind and temperature data, conditions impacting visibility and the presence of dangerous surf warnings [7]. Data regarding distance from the shore, including proximity to the beach and therefore emergency medical service (EMS) intervention, are also helpful to explore factors impacting on surfing and bodyboard-related mortality. Future insights regarding mortality risk related to geomorphological features of various types of surfing locations such as reef breaks and point breaks as compared to beach breaks would be of particular importance to surfers and bodyboarders.

Surfing is one the fastest growing ocean activities [35], with further growth expected with the rise in artificial wave parks [69, 70] and the recent addition of surfing to the 2020 Olympic games. In Australia, the COVID-19 pandemic spurred increased interest in surfing, causing national surfboard shortages as demand increased [71]. Such developments are likely to increase the proportion of novice surfers who may be at an increased risk of injury and death. Further research is also required to explore the risk of drowning and injuries in artificial wave environments.

### 4.1 Strengths and limitations

This study is one of the first studies globally, and the first in Australia to our knowledge, to quantify mortality and exposure measurements of mortality risk among surfers and bodyboarders. This study uses multiple data sources, including coronial data, which provide detailed data on risk factors. The use of both population data and participation data provides an increased understanding of the impact of exposure on mortality risk. There are however, limitations associated with this study. Firstly, the risk factors discussed do not account for other influential factors (e.g. level of experience) as the data does not exist and cannot be provided post-mortem, which may be considered limiting for drawing some conclusions. There may also be cases examined within this study where pre-existing medical conditions were identified at autopsy, however, were not coded as a primary or contributory cause of death. Further, case data (including the contributory medical conditions and causes of death) associated with the included open (i.e., under coronial investigation) coronial cases (n = 8) may change pending the outcome of coronial investigations. Although likely to be minimal, some misclassification of cause of death diagnosis may exist as coroner determination is not always consistent for drowning vs. other causes of deaths, as deaths not drowning-related might have had a trigger before drowning took place (e.g. head trauma with loss of consciousness, re-gained consciousness but were already respiratory-impaired) [72, 73]. Furthermore, drowning is a difficult diagnosis that cannot be confirmed by post-mortem investigation only, as no findings are specific to drowning. Drowning is ruled as the cause of death once other causes are excluded and the circumstances are suggestive, i.e., a body is found in water.

Cardiac conditions are used in this paper as a catch-all term and we do not differentiate between genetic cardiac abnormalities and lifestyle-related cardiac disease. As such, results should be interpreted with caution [73–75]. In addition, a further three cases reported in media and Surf Life Saving Australia’s incident reporting database which could not be found on the National Coronial Information System were also included in the analyses, but lack the associated coronial documentation. This study also focuses on mortality alone due to data availability, but future research that includes both fatal and non-fatal impacts would provide a more general and holistic injury pattern for surfers and bodyboarders and should be considered. While outside the scope of this paper, future research should further consider more complex multivariate analyses that better characterise how multiple dependent variables are related to interacting predictors. This would be especially useful when considering the complex addition of multiple environmental variables.

Valid and generalisable exposure measurements are recognised as being complex [76]. Exposure calculations included all recorded surfing and bodyboarding decedents (aged 14–80 years of age) and used Australian population data for the same age bracket. However, the participation data used survey respondents aged 16+. A limitation of this could be that calculations could be slightly underestimated, however, we expect this impact to be minimal since it covers >99% of cases (n = 1). Similarly, participation data used to calculate exposure is only available for Australian residents for the years 2014–2020. Given that annual variation within the survey respondents is small (see Section 2.1 Data Sources and Variables), we have expected this impact to be minimal, although it still needs to be acknowledged. As such, exposure calculations excluded surfing and bodyboarding-related fatalities of non-residents and, were taken to be a consistent measure applicable to the entire study period. Additionally, there is a lack of exposure-adjusted risk data in surfing for visitors to Australia, who may be very unfamiliar with, or ill-prepared for, the environmental conditions. Future research should also be extended to include the bystander rescues known to be conducted by surfers and bodyboarders. Despite these limitations, this study provides novel analyses with useful insights for those involved in improving safety for surfers and bodyboarders, both in Australia, and globally.

## 5. Conclusions

Despite the potential risks involved, this study has identified surfing and bodyboarding to be relatively safe with considerably lower mortality risk when compared to other coastal activities. The novel insights this study provides using innovative analyses that are practically applicable and useful for surfers, bodyboarders, and safety practitioners. These results particularly highlight the importance of considering exposure time when calculating risk for safety practice and application. This study has identified surfing and bodyboarding as having lower mortality risks than other coastal activities, identified factors that impact on this risk, and have practical applications that inform efforts to improve safety in both activities. This includes the development of targeted risk reduction strategies for surfers and bodyboarders who live away from the coast, with a particular focus on cardiovascular health. Such strategies will potentially work to reduce risk and improve safety efforts for surfers and bodyboarders in Australia and globally.
